# 6-Chloro-7-fluoro-4-oxo-4*H*-chromene-3-carbaldehyde

**DOI:** 10.1107/S1600536814014706

**Published:** 2014-06-25

**Authors:** Yoshinobu Ishikawa

**Affiliations:** a School of Pharmaceutical Sciences, University of Shizuoka, 52-1 Yada, Suruga-ku, Shizuoka 422-8526, Japan

**Keywords:** crystal structure

## Abstract

In the title compound, C_10_H_4_ClFO_3_, a chlorinated and fluorinated 3-formyl­chromone derivative, all atoms are essentially coplanar (r.m.s. = 0.0336 Å for the non-H atoms), with the largest deviation from the least-squares plane [0.062 (2) Å] being for a benzene-ring C atom. In the crystal, mol­ecules are linked through stacking inter­actions [centroid–centroid distance between the benzene and pyran rings = 3.958 (3) Å and inter­planar distance = 3.259 (3) Å], C—H⋯O hydrogen bonds, and short C⋯O contacts [2.879 (3) Å]. Unsymmetrical halogen–halogen inter­actions between the Cl and F atoms [Cl⋯F = 3.049 (3) Å, C—Cl⋯F = 148.10 (9)° and C—F⋯Cl = 162.06 (13)°] are also formed, giving a meandering two-dimensional network along the *a* axis.

## Related literature   

For related structures, see: Ishikawa & Motohashi (2013 ▶); Ishikawa (2014 ▶). For halogen bonding, see: Auffinger *et al.* (2004 ▶); Metrangolo *et al.* (2005 ▶); Wilcken *et al.* (2013 ▶); Sirimulla *et al.* (2013 ▶). For halogen–halogen inter­actions, see: Hathwar & Guru Row (2011 ▶); Metrangolo & Resnati (2014 ▶); Mukherjee & Desiraju (2014 ▶).
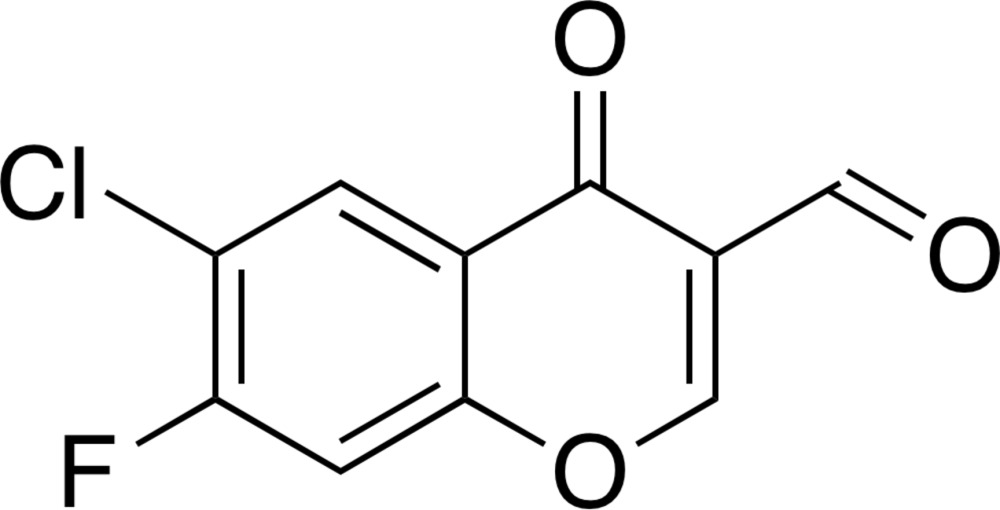


## Experimental   

### 

#### Crystal data  

C_10_H_4_ClFO_3_
*M* *_r_* = 226.59
Orthorhombic, 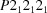

*a* = 5.725 (3) Å
*b* = 32.57 (3) Å
*c* = 4.706 (4) Å
*V* = 877.4 (11) Å^3^
*Z* = 4
Mo *K*α radiation
μ = 0.43 mm^−1^
*T* = 100 K
0.40 × 0.25 × 0.08 mm

#### Data collection  

Rigaku AFC-7R diffractometer
Absorption correction: ψ scan (North *et al.*, 1968 ▶) *T*_min_ = 0.894, *T*_max_ = 0.966
1692 measured reflections
1346 independent reflections
1249 reflections with *F* ^2^ > 2σ(*F* ^2^)
*R*_int_ = 0.009
3 standard reflections every 150 reflections intensity decay: −0.1%

#### Refinement  

*R*[*F* ^2^ > 2σ(*F* ^2^)] = 0.027
*wR*(*F* ^2^) = 0.074
*S* = 1.09
1346 reflections
136 parameters
H-atom parameters constrained
Δρ_max_ = 0.29 e Å^−3^
Δρ_min_ = −0.25 e Å^−3^
Absolute structure: Flack (1983 ▶), 105 Friedel Pairs
Absolute structure parameter: 0.31 (9)

### 

Data collection: *WinAFC Diffractometer Control Software* (Rigaku, 1999 ▶; cell refinement: *WinAFC Diffractometer Control Software*; data reduction: *WinAFC Diffractometer Control Software*; program(s) used to solve structure: *SIR2008* (Burla *et al.*, 2007 ▶); program(s) used to refine structure: *SHELXL97* (Sheldrick, 2008 ▶); molecular graphics: *CrystalStructure* (Rigaku, 2010 ▶); software used to prepare material for publication: *CrystalStructure*.

## Supplementary Material

Crystal structure: contains datablock(s) General, I. DOI: 10.1107/S1600536814014706/zl2593sup1.cif

Structure factors: contains datablock(s) I. DOI: 10.1107/S1600536814014706/zl2593Isup2.hkl

Supporting information file. DOI: 10.1107/S1600536814014706/zl2593Isup3.cml

CCDC reference: 1009489

Additional supporting information:  crystallographic information; 3D view; checkCIF report

## Figures and Tables

**Table 1:** Hydrogen-bond geometry (Å, °)

*D*—H⋯*A*	*D*—H	H⋯*A*	*D*⋯*A*	*D*—H⋯*A*
C7—H3⋯O2^i^	0.95	2.27	3.173 (3)	158
C1—H1⋯O3^ii^	0.95	2.40	3.242 (3)	147

Symmetry codes: (i) 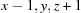; (ii) 
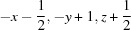
.

**Fig. 1.**
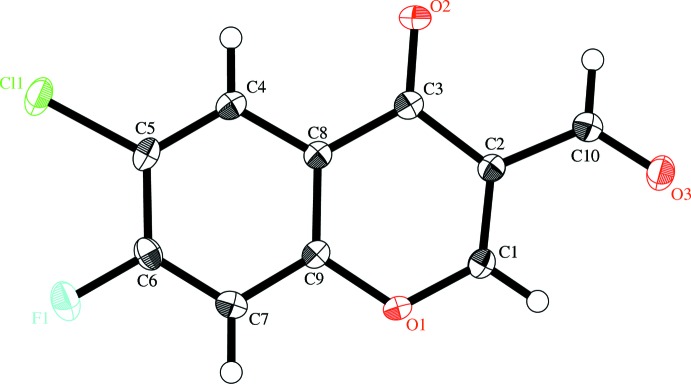
The molecular structure of the title compound, with displacement ellipsoids drawn at the 50% probability level. Hydrogen atoms are shown as small spheres of arbitrary radius.

**Fig. 2.**
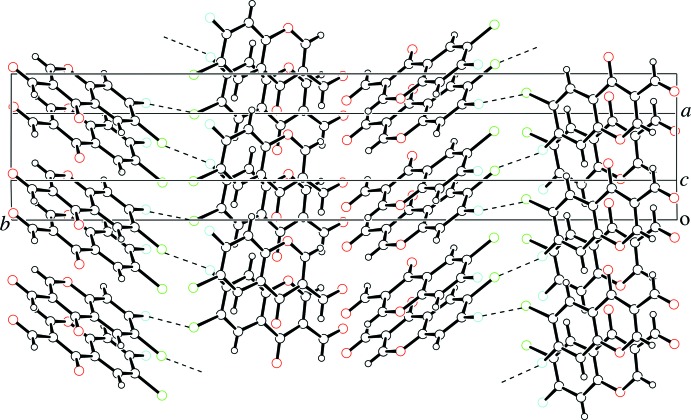
A packing view of the title compound.

**Fig. 3.**
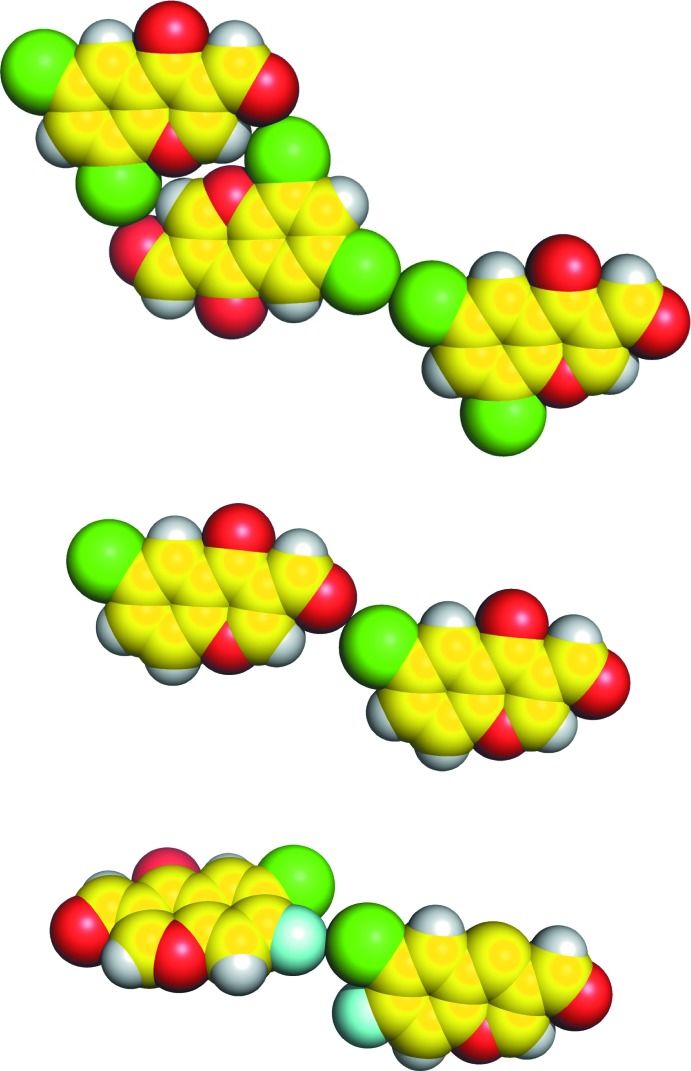
Sphere models of the crystal structures of 6,8-dichloro-4-oxochromene-3-carbaldehyde (top, Ishikawa & Motohashi, 2013), 6-chloro-4-oxo-4H-chromene-3-carbaldehyde (middle, Ishikawa, 2014), and the title compound (bottom).

## supplementary crystallographic information

### S1. Comment

Halogen bonding and halogen···halogen interactions have recently attracted much attention in medicinal chemistry, chemical biology, supramolecular chemistry and crystal engineering (Auffinger *et al.*, 2004, Metrangolo *et al.*, 2005, Wilcken *et al.*, 2013, Sirimulla *et al.*, 2013, Mukherjee *et al.*, 2014, Metrangolo *et al.*, 2014). We have recently reported the crystal structures of chlorinated 3-formylchromone derivatives 6,8-dichloro-4-oxochromene-3-carbaldehyde (Ishikawa & Motohashi, 2013) and 6-chloro-4-oxo-4*H*-chromene-3-carbaldehyde (Ishikawa, 2014). Halogen bonding between the formyl oxygen atom and the chlorine atom at the 8-position and type I halogen···halogen interaction between the chlorine atoms at 6-position are observed in 6,8-dichloro-4-oxochromene-3-carbaldehyde (Fig.3 (top)). On the other hand, a van der Waals contact between the formyl oxygen atom and the chlorine atom at 6-position is found in 6-chloro-4-oxo-4*H*-chromene-3-carbaldehyde (Fig.3 (middle)). As part of our interest in these types of chemical bonding, we herein report the crystal structure of a monochlorinated and monofluorinated 3-formylchromone derivative, 6-chloro-7-fluoro-4-oxo-4*H*-chromene-3-carbaldehyde. The objective of this study is to reveal the inductive effect of the vicinal electron-withdrawing substituent on the chlorine atom at 6-position and the interaction mode(*s*).

The mean deviation of the least-square planes for the non-hydrogen atoms is 0.0336 Å, and the largest deviations is 0.062 (2) Å for C4. These mean that these atoms are essentially coplanar (Fig.1).

In the crystal, the molecules are linked through stacking interaction between the translation-symmetry equivalent^i^ molecules [centroid–centroid distance between the benzene and pyran rings of the 4*H*-chromene units = 3.958 (3) Å, interplanar distance 3.259 (3) Å, i: *x*, *y*, *z* + 1], and through C–H···O hydrogen bonds (see hydrogen bonding table).

A contact between the formyl oxygen atom and the chlorine atom at 6-position is not found in the title compound. Instead, unsymmetrical halogen···halogen interactions are formed between the chlorine and fluorine atoms [Cl1···F1 = 3.049 (3) Å, C5–Cl1···F1 = 148.10 (9)°, C6–F1···Cl1 = 162.06 (13)°] to give a meandering two-dimensional-network along the *a* axis, as shown in Fig.2 and Fig.3 (bottom). It is suggested that the electron-withdrawing substituent at 7-position should make the σ-hole of the chlorine atom at 6-position larger, and the electropositive region of the chlorine atom should contact the electronegative region of the fluorine atom (Hathwar *et al.*, 2011). Symmetrical halogen···halogen interactions (F···F and Cl···Cl) are not observed in the title compound, which might support that the unsymmetrical Cl···F interaction is more favorable than the symmetrical ones.

Furthermore, short contacts between the formyl C10 and O3^ii^ atoms [2.879 (3) Å, ii: –*x* + 1/2, –*y* + 1, *z* + 1/2] are observed. This interesting feature might be caused by strong dipole-dipole interaction between the formyl groups polarized by introduction of the chlorine and fluorine atoms into the chromone ring. These findings should be helpful to understand interaction of halogenated ligands with proteins, and are thus valuable for rational drug design.

### S2. Experimental

5-Chloro-4-fluoro-2-hydroxyacetophenone was prepared from 4-chloro-3-fluorophenol by Fries rearrangement reaction. To a solution of 5-chloro-4-fluoro-2-hydroxyacetophenone (2.4 mmol) in *N*,*N*-dimethylformamide (10 ml) was added dropwise POCl_3_ (6.0 mmol) at 0 °C. After the mixture was stirred for 14 h at room temperature, water (30 ml) was added. The precipitates were collected, washed with water, and dried *in vacuo* (yield: 58%). ^1^H NMR (400 MHz, CDCl_3_): *δ* = 7.36 (d, 1H, *J* = 8.3 Hz), 8.37 (d, 1H, *J* = 8.3 Hz), 8.52 (s, 1H), 10.36 (s, 1H). DART-MS calcd for [C_10_H_4_Cl_1_F_1_O_3_ + H^+^]: 226.991, found 227.014. Single crystals suitable for X-ray diffraction were obtained by slow evaporation of an ethyl acetate/chloroform solution of the title compound at room temperature.

### S3. Refinement

The C(*sp*^2^)-bound hydrogen atoms were placed in geometrical positions [C–H 0.95 Å, *U*_iso_(H) = 1.2*U*_eq_(C)], and refined using a riding model.

### Crystal data

**Table:**

C_10_H_4_ClFO_3_	*F*(000) = 456.00
*M**_r_* = 226.59	*D*_x_ = 1.715 Mg m^−^^3^
Orthorhombic, *P*2_1_2_1_2_1_	Mo *K*α radiation, λ = 0.71069 Å
Hall symbol: P 2ac 2ab	Cell parameters from 25 reflections
*a* = 5.725 (3) Å	θ = 15.0–17.5°
*b* = 32.57 (3) Å	µ = 0.43 mm^−^^1^
*c* = 4.706 (4) Å	*T* = 100 K
*V* = 877.4 (11) Å^3^	Prismatic, yellow
*Z* = 4	0.40 × 0.25 × 0.08 mm

### Data collection

**Table:**

Rigaku AFC-7R diffractometer	*R*_int_ = 0.009
ω scans	θ_max_ = 27.5°
Absorption correction: ψ scan (North *et al.*, 1968)	*h* = −4→7
*T*_min_ = 0.894, *T*_max_ = 0.966	*k* = 0→42
1692 measured reflections	*l* = −3→6
1346 independent reflections	3 standard reflections every 150 reflections
1249 reflections with *F*^2^ > 2σ(*F*^2^)	intensity decay: −0.1%

### Refinement

**Table:**

Refinement on *F*^2^	Hydrogen site location: inferred from neighbouring sites
*R*[*F*^2^ > 2σ(*F*^2^)] = 0.027	H-atom parameters constrained
*wR*(*F*^2^) = 0.074	*w* = 1/[σ^2^(*F*_o_^2^) + (0.0395*P*)^2^ + 0.3855*P*] where *P* = (*F*_o_^2^ + 2*F*_c_^2^)/3
*S* = 1.09	(Δ/σ)_max_ < 0.001
1346 reflections	Δρ_max_ = 0.29 e Å^−^^3^
136 parameters	Δρ_min_ = −0.25 e Å^−^^3^
0 restraints	Absolute structure: Flack (1983), 105 Friedel Pairs
Primary atom site location: structure-invariant direct methods	Absolute structure parameter: 0.31 (9)
Secondary atom site location: difference Fourier map	

### Special details

**Table:**

Refinement. Refinement was performed using all reflections. The weighted *R*-factor (*wR*) and goodness of fit (*S*) are based on *F*^2^. *R*-factor (gt) are based on *F*. The threshold expression of *F*^2^ > 2.0 σ(*F*^2^) is used only for calculating *R*-factor (gt).

### Fractional atomic coordinates and isotropic or equivalent isotropic displacement parameters (Å^2^)

**Table:**

	*x*	*y*	*z*	*U*_iso_*/*U*_eq_	
Cl1	0.39186 (10)	0.273972 (16)	1.18743 (14)	0.02563 (15)	
F1	−0.0630 (3)	0.29836 (4)	1.4050 (3)	0.0262 (4)	
O1	−0.1743 (3)	0.41526 (4)	0.8442 (4)	0.0170 (4)	
O2	0.4433 (3)	0.39789 (5)	0.4232 (4)	0.0197 (4)	
O3	0.0422 (3)	0.49703 (5)	0.2146 (4)	0.0221 (4)	
C1	−0.1015 (4)	0.44135 (6)	0.6415 (5)	0.0164 (5)	
C2	0.1017 (4)	0.43740 (6)	0.4982 (5)	0.0150 (4)	
C3	0.2589 (4)	0.40294 (6)	0.5529 (5)	0.0151 (5)	
C4	0.3078 (4)	0.34031 (6)	0.8557 (5)	0.0166 (5)	
C5	0.2284 (4)	0.31479 (6)	1.0679 (5)	0.0179 (5)	
C6	0.0125 (4)	0.32307 (6)	1.1954 (5)	0.0179 (5)	
C7	−0.1221 (4)	0.35619 (6)	1.1204 (5)	0.0168 (5)	
C8	0.1766 (4)	0.37479 (6)	0.7742 (5)	0.0146 (5)	
C9	−0.0373 (4)	0.38186 (6)	0.9084 (5)	0.0139 (5)	
C10	0.1639 (4)	0.46812 (6)	0.2808 (5)	0.0174 (5)	
H1	−0.1991	0.4640	0.5960	0.0197*	
H2	0.4520	0.3346	0.7640	0.0200*	
H3	−0.2678	0.3614	1.2100	0.0202*	
H4	0.3094	0.4650	0.1861	0.0208*	

### Atomic displacement parameters (Å^2^)

**Table:**

	*U*^11^	*U*^22^	*U*^33^	*U*^12^	*U*^13^	*U*^23^
Cl1	0.0256 (3)	0.0191 (3)	0.0322 (3)	0.0053 (3)	−0.0045 (3)	0.0062 (3)
F1	0.0290 (8)	0.0242 (7)	0.0256 (8)	−0.0050 (7)	−0.0003 (7)	0.0083 (6)
O1	0.0134 (7)	0.0183 (7)	0.0194 (8)	0.0035 (6)	0.0028 (7)	0.0013 (7)
O2	0.0160 (8)	0.0231 (8)	0.0199 (8)	0.0032 (7)	0.0050 (8)	0.0007 (7)
O3	0.0219 (8)	0.0194 (7)	0.0250 (9)	0.0017 (7)	−0.0026 (8)	0.0047 (7)
C1	0.0170 (10)	0.0144 (9)	0.0180 (11)	0.0007 (8)	−0.0019 (11)	−0.0003 (9)
C2	0.0160 (10)	0.0143 (9)	0.0146 (10)	0.0003 (9)	−0.0007 (10)	−0.0016 (8)
C3	0.0150 (10)	0.0159 (10)	0.0144 (10)	−0.0004 (9)	−0.0025 (10)	−0.0029 (9)
C4	0.0161 (10)	0.0170 (10)	0.0169 (11)	0.0015 (9)	−0.0019 (10)	−0.0031 (9)
C5	0.0193 (11)	0.0136 (10)	0.0207 (11)	0.0023 (9)	−0.0049 (10)	−0.0004 (9)
C6	0.0208 (11)	0.0168 (10)	0.0162 (11)	−0.0050 (9)	−0.0007 (11)	0.0007 (10)
C7	0.0152 (10)	0.0202 (10)	0.0150 (10)	−0.0022 (9)	0.0002 (10)	−0.0025 (9)
C8	0.0157 (10)	0.0142 (9)	0.0137 (10)	−0.0001 (8)	−0.0013 (9)	−0.0026 (9)
C9	0.0137 (10)	0.0134 (9)	0.0147 (10)	−0.0004 (8)	−0.0034 (9)	−0.0029 (9)
C10	0.0175 (10)	0.0191 (10)	0.0156 (10)	−0.0009 (9)	−0.0010 (10)	−0.0013 (9)

### Geometric parameters (Å, º)

**Table:**

Cl1—C5	1.720 (3)	C4—C5	1.377 (4)
F1—C6	1.344 (3)	C4—C8	1.405 (3)
O1—C1	1.344 (3)	C5—C6	1.400 (4)
O1—C9	1.375 (3)	C6—C7	1.372 (3)
O2—C3	1.231 (3)	C7—C9	1.389 (3)
O3—C10	1.212 (3)	C8—C9	1.397 (3)
C1—C2	1.351 (4)	C1—H1	0.950
C2—C3	1.461 (3)	C4—H2	0.950
C2—C10	1.474 (3)	C7—H3	0.950
C3—C8	1.465 (3)	C10—H4	0.950
			
Cl1···F1	2.908 (2)	F1···H3	2.5356
F1···C9	3.589 (4)	O1···H3	2.5157
O1···C3	2.862 (3)	O2···H2	2.6126
O1···C6	3.590 (4)	O2···H4	2.5720
O2···C1	3.576 (4)	O3···H1	2.5075
O2···C4	2.874 (3)	C1···H4	3.2741
O2···C10	2.870 (3)	C3···H1	3.2973
O3···C1	2.828 (3)	C3···H2	2.6768
C1···C7	3.576 (4)	C3···H4	2.6745
C1···C8	2.761 (4)	C5···H3	3.2890
C2···C9	2.763 (4)	C6···H2	3.2547
C4···C7	2.806 (4)	C8···H3	3.2967
C5···C9	2.766 (4)	C9···H1	3.1900
C6···C8	2.766 (4)	C9···H2	3.2681
Cl1···F1^i^	3.380 (3)	C10···H1	2.5568
Cl1···F1^ii^	3.049 (3)	H1···H4	3.4924
F1···Cl1^iii^	3.380 (3)	Cl1···H2^v^	3.3732
F1···Cl1^iv^	3.049 (3)	Cl1···H3^i^	3.4511
F1···C4^v^	3.297 (3)	F1···H2^vi^	3.4577
F1···C5^v^	3.577 (4)	F1···H2^v^	3.5970
F1···C8^v^	3.331 (3)	O1···H2^iii^	3.4090
O1···O2^iii^	3.006 (3)	O1···H3^viii^	3.5035
O1···O2^vi^	3.540 (3)	O1···H4^v^	3.5895
O1···O3^v^	3.416 (3)	O2···H1^i^	3.0802
O1···C2^v^	3.533 (4)	O2···H3^vii^	2.2707
O1···C3^iii^	3.545 (3)	O3···H1^viii^	3.3969
O1···C10^v^	3.307 (3)	O3···H1^xi^	2.4042
O2···O1^vii^	3.540 (3)	O3···H4^ix^	2.9039
O2···O1^i^	3.006 (3)	O3···H4^x^	2.6777
O2···C1^i^	3.138 (3)	C1···H3^viii^	3.4372
O2···C4^viii^	3.354 (4)	C1···H4^v^	3.5633
O2···C5^viii^	3.411 (4)	C1···H4^x^	3.4837
O2···C7^vii^	3.173 (3)	C2···H3^viii^	3.5279
O2···C8^viii^	3.496 (4)	C2···H4^v^	3.5642
O3···O1^viii^	3.416 (3)	C2···H4^x^	3.3373
O3···O3^ix^	3.352 (3)	C3···H3^vii^	3.4320
O3···O3^x^	3.352 (3)	C4···H3^i^	3.0253
O3···C1^viii^	3.353 (4)	C5···H2^v^	3.5757
O3···C1^xi^	3.242 (3)	C5···H3^i^	3.3268
O3···C2^ix^	3.123 (3)	C7···H2^iii^	3.0416
O3···C3^ix^	3.534 (4)	C9···H2^iii^	3.3732
O3···C10^ix^	2.879 (3)	C10···H1^xi^	3.5669
O3···C10^x^	3.349 (4)	C10···H4^ix^	3.5488
C1···O2^iii^	3.138 (3)	C10···H4^x^	2.8982
C1···O3^v^	3.353 (4)	H1···O2^iii^	3.0802
C1···O3^xii^	3.242 (3)	H1···O3^v^	3.3969
C1···C10^v^	3.481 (4)	H1···O3^xii^	2.4042
C2···O1^viii^	3.533 (4)	H1···C10^xii^	3.5669
C2···O3^x^	3.123 (3)	H1···H1^xi^	3.3725
C2···C7^viii^	3.435 (4)	H1···H1^xii^	3.3725
C2···C9^viii^	3.407 (4)	H1···H4^iii^	3.4116
C3···O1^i^	3.545 (3)	H1···H4^x^	3.2403
C3···O3^x^	3.534 (4)	H2···Cl1^viii^	3.3732
C3···C6^viii^	3.404 (4)	H2···F1^viii^	3.5970
C3···C7^viii^	3.349 (4)	H2···F1^vii^	3.4577
C3···C9^viii^	3.542 (4)	H2···O1^i^	3.4090
C4···F1^viii^	3.297 (3)	H2···C5^viii^	3.5757
C4···O2^v^	3.354 (4)	H2···C7^i^	3.0416
C4···C6^viii^	3.581 (4)	H2···C9^i^	3.3732
C4···C7^i^	3.531 (4)	H2···H3^vii^	3.1828
C5···F1^viii^	3.577 (4)	H2···H3^i^	2.7815
C5···O2^v^	3.411 (4)	H3···Cl1^iii^	3.4511
C6···C3^v^	3.404 (4)	H3···O1^v^	3.5035
C6···C4^v^	3.581 (4)	H3···O2^vi^	2.2707
C6···C8^v^	3.337 (4)	H3···C1^v^	3.4372
C7···O2^vi^	3.173 (3)	H3···C2^v^	3.5279
C7···C2^v^	3.435 (4)	H3···C3^vi^	3.4320
C7···C3^v^	3.349 (4)	H3···C4^iii^	3.0253
C7···C4^iii^	3.531 (4)	H3···C5^iii^	3.3268
C7···C8^v^	3.571 (4)	H3···H2^iii^	2.7815
C8···F1^viii^	3.331 (3)	H3···H2^vi^	3.1828
C8···O2^v^	3.496 (4)	H4···O1^viii^	3.5895
C8···C6^viii^	3.337 (4)	H4···O3^ix^	2.6777
C8···C7^viii^	3.571 (4)	H4···O3^x^	2.9039
C9···C2^v^	3.407 (4)	H4···C1^viii^	3.5633
C9···C3^v^	3.542 (4)	H4···C1^ix^	3.4837
C9···C10^v^	3.506 (4)	H4···C2^viii^	3.5642
C10···O1^viii^	3.307 (3)	H4···C2^ix^	3.3373
C10···O3^ix^	3.349 (4)	H4···C10^ix^	2.8982
C10···O3^x^	2.879 (3)	H4···C10^x^	3.5488
C10···C1^viii^	3.481 (4)	H4···H1^i^	3.4116
C10···C9^viii^	3.506 (4)	H4···H1^ix^	3.2403
C10···C10^ix^	3.289 (4)	H4···H4^ix^	3.3442
C10···C10^x^	3.289 (4)	H4···H4^x^	3.3442
Cl1···H2	2.8259		
			
C1—O1—C9	118.64 (17)	C3—C8—C4	121.5 (2)
O1—C1—C2	124.15 (19)	C3—C8—C9	120.02 (19)
C1—C2—C3	121.03 (19)	C4—C8—C9	118.5 (2)
C1—C2—C10	119.31 (19)	O1—C9—C7	115.77 (19)
C3—C2—C10	119.7 (2)	O1—C9—C8	122.08 (19)
O2—C3—C2	122.9 (2)	C7—C9—C8	122.1 (2)
O2—C3—C8	123.02 (19)	O3—C10—C2	124.5 (2)
C2—C3—C8	114.06 (19)	O1—C1—H1	117.920
C5—C4—C8	120.3 (2)	C2—C1—H1	117.932
Cl1—C5—C4	121.60 (18)	C5—C4—H2	119.879
Cl1—C5—C6	119.27 (17)	C8—C4—H2	119.849
C4—C5—C6	119.1 (2)	C6—C7—H3	121.234
F1—C6—C5	118.87 (19)	C9—C7—H3	121.245
F1—C6—C7	118.6 (2)	O3—C10—H4	117.735
C5—C6—C7	122.5 (2)	C2—C10—H4	117.738
C6—C7—C9	117.5 (2)		
			
C1—O1—C9—C7	179.81 (16)	C8—C4—C5—Cl1	−176.25 (17)
C1—O1—C9—C8	1.4 (3)	C8—C4—C5—C6	1.7 (3)
C9—O1—C1—C2	−1.3 (3)	H2—C4—C5—Cl1	3.7
C9—O1—C1—H1	178.7	H2—C4—C5—C6	−178.3
O1—C1—C2—C3	1.1 (4)	H2—C4—C8—C3	−1.2
O1—C1—C2—C10	−179.67 (17)	H2—C4—C8—C9	178.6
H1—C1—C2—C3	−178.9	Cl1—C5—C6—F1	−1.2 (3)
H1—C1—C2—C10	0.3	Cl1—C5—C6—C7	176.87 (14)
C1—C2—C3—O2	178.64 (19)	C4—C5—C6—F1	−179.17 (19)
C1—C2—C3—C8	−0.9 (3)	C4—C5—C6—C7	−1.1 (4)
C1—C2—C10—O3	−1.5 (4)	F1—C6—C7—C9	178.33 (16)
C1—C2—C10—H4	178.5	F1—C6—C7—H3	−1.7
C3—C2—C10—O3	177.79 (19)	C5—C6—C7—C9	0.3 (4)
C3—C2—C10—H4	−2.2	C5—C6—C7—H3	−179.7
C10—C2—C3—O2	−0.6 (3)	C6—C7—C9—O1	−178.42 (18)
C10—C2—C3—C8	179.86 (17)	C6—C7—C9—C8	−0.0 (3)
O2—C3—C8—C4	1.2 (4)	H3—C7—C9—O1	1.6
O2—C3—C8—C9	−178.51 (18)	H3—C7—C9—C8	180.0
C2—C3—C8—C4	−179.22 (17)	C3—C8—C9—O1	−1.3 (3)
C2—C3—C8—C9	1.0 (3)	C3—C8—C9—C7	−179.63 (17)
C5—C4—C8—C3	178.79 (18)	C4—C8—C9—O1	178.90 (18)
C5—C4—C8—C9	−1.5 (3)	C4—C8—C9—C7	0.6 (3)

Symmetry codes: (i) *x*+1, *y*, *z*; (ii) *x*+1/2, −*y*+1/2, −*z*+3; (iii) *x*−1, *y*, *z*; (iv) *x*−1/2, −*y*+1/2, −*z*+3; (v) *x*, *y*, *z*+1; (vi) *x*−1, *y*, *z*+1; (vii) *x*+1, *y*, *z*−1; (viii) *x*, *y*, *z*−1; (ix) −*x*+1/2, −*y*+1, *z*−1/2; (x) −*x*+1/2, −*y*+1, *z*+1/2; (xi) −*x*−1/2, −*y*+1, *z*−1/2; (xii) −*x*−1/2, −*y*+1, *z*+1/2.

### Hydrogen-bond geometry (Å, º)

**Table:**

*D*—H···*A*	*D*—H	H···*A*	*D*···*A*	*D*—H···*A*
C7—H3···O2^vi^	0.95	2.27	3.173 (3)	158
C1—H1···O3^xii^	0.95	2.40	3.242 (3)	147

Symmetry codes: (vi) *x*−1, *y*, *z*+1; (xii) −*x*−1/2, −*y*+1, *z*+1/2.
